# From Pandemic to Progression: An Educational Framework for the Implementation of Virtual Mental Healthcare for Children and Youth as a Response to COVID-19

**DOI:** 10.1007/s10879-020-09478-0

**Published:** 2020-10-23

**Authors:** Bridget T. Doan, Yue Bo Yang, Erin Romanchych, Seena Grewal, Suneeta Monga, Tony Pignatiello, Pier Bryden, Chetana Kulkarni

**Affiliations:** 1 Hospital for Sick Children (SickKids), Toronto, ON Canada; 2 MD Undergraduate Program, University of British Columbia, Vancouver, BC Canada; 3 University of Toronto, Toronto, ON Canada

**Keywords:** Mental health, Virtual care, Covid-19, Children, Youth, Framework

## Abstract

COVID-19 restrictions have necessitated child/youth mental health providers to shift towards virtually delivering services to patients’ homes rather than hospitals and community mental health clinics. There is scant guidance available for clinicians on how to address unique considerations for the virtual mental healthcare of children and youth as clinicians rapidly shift their practices away from in-person care in the context of the COVID-19 pandemic. Therefore, we bridge this gap by discussing a six-pillar framework developed at Hospital for Sick Children (SickKids) in Toronto, Ontario, Canada, for delivering direct to patient virtual mental healthcare to children, youth and their families. We also offer a discussion of the advantages, disadvantages, and future implications of such services.

## Introduction

The teaching and application of child and youth telemental health has occurred across diverse settings (Pignatiello et al. 2009; Serhal et al. 2017; Thomas et al. 2018), typically showing similar parental satisfaction, diagnostic profiles, treatment adherence, and improvement in primary outcomes compared to in-person visits (AACAP 2017; Myers and Cain 2008). Traditionally, telemental health has been delivered in clinical settings with a technologically and medically optimized environment where healthcare providers, medical services, and interventions may be readily available in the case of safety or general medical concerns (AACAP 2017), often with a goal to provide access to underserviced populations. More recent data demonstrates opportunities for providing telemental healthcare in non-clinical settings such as schools, residential treatment centers, correctional facilities and even patients' homes (Myers et al. 2017).

In response to the COVID-19 pandemic, clinicians who may not have regularly practiced telemental healthcare previously have been catalyzed to transition rapidly to delivering telemental health services directly to patients’ homes (Fagiolini et al. 2020), with clinicians, including trainees, also often connecting from their own homes. We define this newly catalyzed practice as direct to patient virtual mental healthcare (VMHC) hereafter. This rapid transition to VMHC presents unique considerations for clinicians, trainees and patients/families including extensive variation in access to technologies, availability of a responsible adult (when seeing children & some youth), and private assessment spaces. The onus is on clinicians and trainees, who may include medical students, residents, and graduate students, to optimize the encounter with consideration of the patient’s available resources, socioeconomic status, stability of housing, and relationship with the adults present. Maheu and colleagues (2017) have developed an interdisciplinary framework of domains of competency for delivering telepsychology, including considerations for clinical care, technology, legal and ethical issues, and the virtual environment. Building on the existing literature and recognizing the unique considerations in providing VMHC to children, youth, and their families, we have created a six-pillar framework on how to optimally deliver VMHC to children and youth in an academic teaching hospital. Furthermore, we speculate that as patients become accustomed to these services, there may be an impetus to continue to offer VMHC on an ongoing basis following the eventual resolution of the COVID-19 pandemic, making such a framework an invaluable tool for clinicians and trainees providing mental healthcare to this population.

## Framework Development

This framework for clinicians and trainees was developed in response to needs associated with the COVID-19 pandemic and its resulting restrictions. A series of virtual meetings of multi-disciplinary mental health clinicians from The Hospital for Sick Children (SickKids), associated with the University of Toronto, took place over a two-week period to plan for rapid transition of in-person care to VMHC. This framework was informed by a Plan-Do-Study-Act (Gillam and Siriwardena 2013) approach, as well as consultation with experts in conducting virtual healthcare and review of the limited available literature.

## Virtual Mental Healthcare Framework (Fig. 1)

**Fig. 1:**
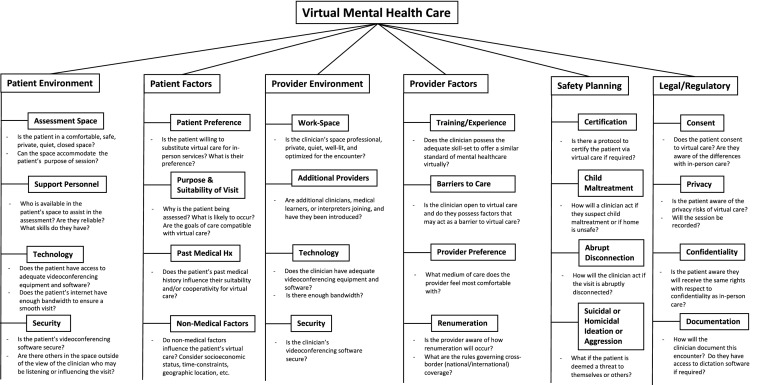
A six-pillar framework for providing virtual mental healthcare to children, youth & families

### Patient and Caregiver Environment

In VMHC, assessment spaces will vary. We suggest patients, where possible, reserve a private, quiet, safe, and comfortable space within their homes that also satisfies the purpose of assessment or treatment (Myers and Cain 2008). For example, if the visit requires assessment of a toddler, the room should be large enough to accommodate play and be equipped with necessary toys and books. Patients may only have access to sub-optimal spaces, in which case flexibility and creative problem solving is necessary. The combined use of headphones and typing text may allow for a private discussion to compensate for use of a space that may not otherwise be considered private. Other creative ways that patients and families have used to find privacy include completing sessions in their washroom, their garage, or even a parked vehicle (without the use of bluetooth/vehicle speakers for privacy reasons), where they are less likely be disturbed or overheard. In addition to the physical space, enhancing the therapeutic space between patients and families with the clinicians (e.g., minimizing distractions and interruptions) can help to foster the therapeutic relationship (Maheu et al. 2017). Early communication between patient/family and clinician is therefore necessary to ensure the highest standard of care possible.

Families should also be aware that a responsible adult support person in the home may need to act as an assessment-support and emergency safety contact. Likely, this is the parent, legal guardian, or another responsible adult identified by the patient. For young children, such as pre-schoolers or early school-agers, the responsible adult may help re-direct the child’s attention back to the screen. This adult may help administer aspects of a mental status examination or play a pivotal role safety planning. Ideally, the responsible adult should also have a relationship with the patient that allows the child/youth to feel safe, trusting, and be able to support honesty with the clinician (Shore et al. 2018).

Access to adequate hardware, software, and bandwidth that allows seamless communication with providers is another consideration in VMHC. Sufficient camera and screen resolutions, the ability to clearly transmit verbal and non-verbal communication with minimal audio or visual delays, and cameras that can pan and tilt to capture images of children who move around is recommended when possible (Ontario Health, Quality Division). A stationary computer and webcam should be utilized; where these are not available, a stationary mobile phone or tablet on a stand can be used.

Finally, the patient’s privacy regarding their personal health information should replicate an in-person visit. Patients and families should receive instructions concerning secure video conferencing software and should strive for complete privacy within their home, so that they are able to be open and honest during assessments (Ontario Health, Quality Division). Prior to the virtual visit, all of the above should be reviewed verbally with the patient and/or responsible adult, and recommendations provided in written format, either by the clinician or by the administrative staff.

### Patient and Caregiver Factors

Patients must be willing and able to engage in virtual mental healthcare. Furthermore, both the purpose and suitability of the visit must be compatible with using the virtual modality (Myers and Cain 2008). Unwillingness may represent a multitude of factors such as personal preference, safety, communication limitations, home environment and privacy. If patients have reservations, their reasons should be explored, and a decision as to whether to proceed with VMHC should be informed by the patient’s preferences, cultural expectations, and experiences with technology (Maheu et al. 2017). Clinicians should be mindful that youth with social anxiety or body image concerns may struggle with video conferencing. In such cases it may be of value to allow the youth to participate off camera or with audio only, with a goal of increasing comfort or willingness to be present on camera. Consideration should also be made of a patient’s potential for violence towards themselves or others, or other risks. For example, if the patient is likely to require psychiatric admission, their assessment might be ideally conducted in a facility where emergency services are readily available. For youth with communication, visual or hearing difficulties, creativity is required. If possible, written responses via a chat feature may facilitate communication. Children and adolescents with neurodevelopmental disorders and younger children may feel more comfortable in their own homes but may also be easily distracted, making the presence of a responsible adult essential. Having the adult prepare the space with activities the patient enjoys or comfort items, can assist in the success of the virtual experience. Clinicians should consider non-medical factors which may influence virtual care. Will the patient be able to communicate openly? Will the visit save time for vulnerable patients or families who might otherwise miss academic or employment opportunities? Are patients seeking virtual care to avoid prohibitive costs related to travel? Is it safe for the family to leave the home or does this pose a health risk to them or others? It is important to note interpreter services can be provided virtually and language should therefore not be a barrier. These factors, patient context, and the goals of care should be considered holistically in a risk and benefit analysis to determine whether VMHC is a suitable modality of care.

### Provider Environment

The clinician and trainee’s spaces should be tidy, quiet, well-lit, private, and meet recommended professional standards for clinical spaces (Shore et al. 2018). If available, headsets should be used to maintain the highest standard of privacy (Ontario Health, Quality Division). Those providing virtual care should assume they are “always-on” when in the vicinity of the equipment and should keep their microphone on mute when not speaking to minimize background noise on the patient’s side (Ontario Health, Quality Division). If possible, all participants joining remotely (including interpreters and others involved in care who may be joining the meeting) can be kept in the virtual waiting room until the primary clinicians (staff and/or trainee) have greeted the patient and any adult(s) present. This process should be planned ahead of time so all are aware of what will occur. All participants should be introduced with their name and role at the start of the visit (Myers and Cain 2008; Shore et al. 2018). If possible, we recommend all individuals show their ID badges by holding them to the camera. This is particularly important should the family be meeting a clinician with whom they do not have a pre-existing relationship. Clinicians must have access to adequate software, hardware, and internet bandwidth (recommended 800kpbs - 1.8Mbps up/down) to ensure high quality screen and camera resolution, and acceptable audio-visual synchronization to maintain a smooth encounter (Shore et al. 2018). When possible, ethernet or a similar alternative should be used to increase quality and reliability of the videoconferencing. Access to technical support to troubleshoot challenges will increase clinician confidence. The use of a secure end-to-end encryption network is preferred, and video conferencing platforms should ideally be endorsed and supported by the clinician’s mental health organization, institution, or regulatory body (Ontario Health, Quality Division). The use of 3rd party video conferencing software not endorsed by these bodies should be used only as a last resort due to security and privacy concerns (Ontario Health, Quality Division).

### Provider Factors

Willingness and flexibility on the part of the clinician to embrace technology and new models of care are important, particularly when physical distancing is critical. In fact, using and understanding technology as it applies to VMHC can be considered an area of clinical competency, which all clinicians are encouraged to continuously refine and develop (Maheu et al. 2017). At our institution, all clinicians, including trainees, have access to a protocol with safety guidelines to support their practice, which has been essential. Clinicians can build on their experience and knowledge from in-person care to form a strong therapeutic alliance virtually although modifications may be required (Myers and Cain 2008; Shore et al. 2018). For example, clinicians may need to be more directive in a virtual visit, especially if managing multiple people in the room. Body language and positioning should be tailored for virtual visits: leaning slightly forward toward the camera can convey caring and empathy (Myers and Cain 2008), and deliberate hand gestures can be used to build therapeutic alliance (e.g., virtual handshake or “high 5”). Flexibility and the ability to tolerate disruptions is an asset in virtual care. Reflection on personal barriers to delivering virtual care, such as relative discomfort with technology, will allow clinicians to seek support to address these concerns. It is helpful for clinicians to become familiar with tools such as screen sharing, whiteboard, and chat features, which can engage patients, maintain privacy and be used for teaching. Given differing comfort levels, training, and expertise, trainees may require specific supervision focused on developing rapport, adapting therapeutic techniques, and managing decreased non-verbal cues in the virtual setting. Trainee supervision should occur synchronously when possible to encourage exposure to the platform and an opportunity to troubleshoot as needed. If available, trainees and supervisors can use the private chat feature during the session to provide guidance and support. If the virtual platform allows, the waiting room feature can be used for real time supervision or team discussions. The use of a “one-way mirror” for observation can be mimicked by having audio and video off after introductions are completed. Similarly, when audio is muted but video remains on, this can be considered analogous to the trainee and supervisor being together in the same physical space as the patient.

### Safety Planning

As with in-person care, a safety protocol to address potential patient risks is essential for all clinicians. At our institution, all patients and responsible adults are emailed a VMHC introduction letter. This letter reviews the roles of the patient and responsible adult including the need for an adult to be accessible for the duration of the appointment in the event of any acute or imminent safety concerns. Participants are informed and must consent to our protocol, specifically, the responsible adult may be asked to help transport the patient safely to hospital for further care. If suicidal, homicidal or violent ideation is identified, clinicians are required to virtually conduct a risk assessment. Such an assessment may lead to safety planning such that the patient can remain in the home, or a determination that the patient will need to be brought to hospital for further in-person assessment and possible admission. Clinicians should be prepared to certify patients virtually if deemed necessary, which may involve the use of an emergency contact or local authorities. Depending on local regulations, clinicians and trainees may need/be permitted to fax copies of their request for psychiatry emergency assessment to local police stations or emergency departments (Myers and Cain 2008). Local regulations will determine the details of how best to proceed in these circumstances. As with in-person care, identification of imminently concerning child maltreatment necessitates immediate notification of the local child protection agency. Lastly, in the event of an abrupt disconnection from the virtual visit, either voluntarily or involuntarily, clinicians should re-connect with the patient or responsible adult as soon as possible to ensure safety and further work-up if needed. In these situations, it is essential to have ready access to alternate contact information for the patient and their responsible adult support person. If unable to reach the patient or responsible adult, the clinician should contact local authorities if there is a safety concern. If learners are managing the session independently, their supervisor should be immediately contacted so they can help manage the situation.

### Legal and Regulatory

Prior to engaging in VMHC, clinicians have a responsibility to adhere to interprofessional and discipline-specific professional standards, as well as federal and local laws and regulations pertaining to their clinical practice (e.g., consent, documentation, legal-technology risk factors) (Maheu et al. 2017). Many regulatory systems have made rapid changes to their policies in response to the pandemic, thus making it critical that clinicians remain appraised of such changes (“Telemedicine and Virtual Care Guidelines (and other clinical resources for COVID-19)”). Documentation of verbal consent and acceptance of virtual care by the patient and responsible adult, in conjunction with ensuring the responsible adult is aware and understands the potential security flaws, risks, benefits, and differences from in-person care, are needed at the start of the encounter (Appendix A). Additionally, the responsible adult must understand there is a potential need for the patient to subsequently be further assessed in-person. If parts of the session will be recorded for assessment, teaching, or research purposes, patients must be aware of this and offered the ability to decline recording (Shore et al. 2018). At our institution, we have disabled the recording, whiteboard, and file sharing features for all participants and only the clinician can screenshare in our video conferencing platform to further protect privacy. It is important to note some commercial video conferencing platforms offer different versions of their products, with some meeting health care security and privacy needs while others do not. As such, it is essential that videoconferencing platforms are approved by all appropriate regulatory systems including the clinician's mental health organization/hospital and professional regulatory bodies. Patients should understand that depending on the software used, differing levels of privacy risks may be present. As noted above, patients are entitled to the same confidentiality rights as in-person care, and clinicians should similarly document the encounter in their health records.

## Discussion

Here, we propose an initial six-pillar framework for provision of VMHC to children and youth aimed to support clinicians, including trainees, within this new care requirement. It encourages the clinician to consider patient, caregiver, and provider factors to maximize the success of VMHC. The COVID-19 pandemic has forced the delivery of VMHC to prominence (Fagiolini et al. 2020), illuminating many advantages and some disadvantages compared to traditional telemental health and/or in-person services. Time, cost, staff efficiency, convenience, and the ability to view patients in multiple environments, are all benefits. Although there can be a learning curve for clinicians, as we note at our site, even clinicians who initially struggled with the technological aspects quickly become proficient with adequate support and training. Moreover, given that traditional telemental health has been well studied with demonstrated comparable outcomes to in-person visits (AACAP 2017; Myers and Cain 2008), it is plausible that VMHC can yield similar outcomes.

However, we acknowledge that VMHC is an emerging area with scant evidence or guidelines available for working with children and youth. Given its relative novelty, there are many considerations that must addressed, including issues of equity/access, homelessness, and unsafe living environments that challenge the delivery of VMHC. Ensuring internet infrastructure in underserved communities, including areas more likely to be populated by racialized families, will be critical for ensuring access to VMHC. These and other details can only be elucidated with time, collaboration, and continued research, which we predict will occur at an accelerated pace as efforts at physical distancing continue to become integrated into daily life. Furthermore, an increased demand in child and youth mental health resources is occuring as patients continue to experience disruption in their regular routines (including return to school), compounded by a decrease in social supports (Golberstein et al. 2020). Interestingly, while telemental health has been actively practiced by a select group of clinicians for over 30 years, the current pandemic seems to have propelled the use of technology by a much wider group of clinicians to deliver mental healthcare. Most supervisors and trainees are learning together about this new modality. Training in best practices will be important and will continue to evolve. A well thought out approach to VMHC may also provide opportunities to address inequities in access to care that may be related to geography, access to technology, systemic racism or other barriers. We speculate that as both patients and clinicians become accustomed to VMHC, the delivery of these services will increase in a diversity of clinical settings, thus reforming the teaching and delivery of post-pandemic mental healthcare.

## Appendix A

Author confirmed that they and (patient name) and parent/guardian/responsible adult received the Virtual Mental Healthcare Orientation Letter. This document outlines the expectation that an adult is present at the same location as the child/adolescent during the appointment (unless alternative plan was previously discussed), provides an emergency contact number, and agreed to responsibilities in the event of an acute safety concern (i.e., to bring the child/adolescent to the nearest emergency department if requested, or to remain with the patient if emergency services are required). Potential risks of virtual care with respect to privacy and security were discussed. Child/adolescent and parent/guardian/responsible adult agreed to these parameters. Informed verbal consent was obtained to communicate and provide care via a virtual appointment.
